# The data of genomic and phenotypic profiles of the N-acyl homoserine lactone-producing algicidal bacterium *Stenotrophomonas rhizophila* GA1

**DOI:** 10.1016/j.dib.2018.10.051

**Published:** 2018-10-23

**Authors:** Panqing Yin, Qin Zhang, Jianming Zhu, Guoqiang Wu, Sanjun Yin, Zhenhua Ma, Jin Zhou

**Affiliations:** a The School of Life Science and Engineering, Lanzhou University of Technology, Lanzhou 730050, Gansu Province, PR China; b Jiangxi University of Finance and Economics, 330013 Jiangxi Province, PR China; c The Devision of Ocean Science and Technology, Graduate School at Shenzhen, Tsinghua University, Shenzhen 518055, Guangdong Province, PR China; d Hengchuang Genetic Biotech Co., Ltd, Shenzhen 518055, Guangdong Province, PR China

## Abstract

Herein, an algicidal strain, *Stenotrophomonas rhizophila* GA1, was isolated from a marine dinoflagellate and its genome was sequenced using next-generation sequencing technology. The genome size of *S. rhizophila* GA1 was determined to be 5.92 Mb with a G+C content of 62.39%, comprising eight scaffolds of 67 contigs. A total of 4579 functional proteins were assigned according to COG categories. *In silico* genome annotation protocols identified multiple putative LuxI-like genes located in the upstream position at contig 4. The thin-layer chromatography analysis showed that three kinds of acyl homoserine lactone (AHL) signals could be produced by *S. rhizophila* GA1. This work describes an algicidal bacterium capable of generating AHL molecules for its ecological adaptation. The annotated genome sequence of this strain may represent a valuable tool for studying algae-bacterium interactions and developing microbial methods to control harmful algae. The genome scaffolds generated are available in the National Center Biotechnology Information (NCBI) BioProject with accession number PRJNA485554.

**Specifications table**

**Table:** 

Subject area	Biology
More specific subject area	Bacteriology, Genomics, Ecology
Type of data	Figures, Tables
How data was acquired	The whole genome was sequenced with an Illumina Hi-Seq. 2500
Data format	Analyzed
Experimental factors	Isolation and characterization of native strains *S. rhizophila* GA1. Genomic DNA, extraction and sequencing procedures.
Experimental features	Genome of the *S. rhizophila* GA1 was sequenced and assembled.
Data source location	The strain was isolated from the dinoflagellate (*Gymnodinium aerucyinosum*) on the Shenzhen coast of China (22°59′42.19″N, 114°54′74.01″E).
Data accessibility	Data is with this article. Also, the whole-genome of *S. rhizophila* GA1 has been deposited in the GenBank database under accession numbers PRJNA485554 (https://www.ncbi.nlm.nih.gov/bioproject/?term=PRJNA485554).
Related research article	None

**Value of the data**

• It is the first whole genome of algicidal bacterium *S. rhizophila* GA1.
• This data allows other researchers to extend the study about the algae-bacterium interactions.
• The data could be help us developing microbial methods to control harmful algae.

## 1 Data

**Fig. 1:**
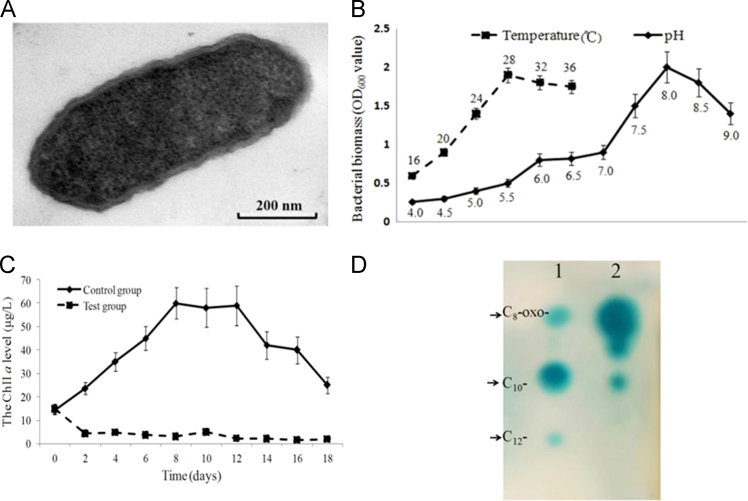
(A) Electron micrographs of cells of *S. rhizophila* GA1. Preparation and electron microscopy conditions were as described by Hahnke et al. [10]. Magnification: 50,000×. (B) Optimal temperature and pH conditions for the growth of *S. rhizophila* GA1. Error bars indicate the mean value ± standard deviation of three optical density measurements at 600 nm (OD_600_). (C) Algicidal activity of *S. rhizophila* GA1. The control group was sole-cultured *G. aerucyinosum*, and the initial concentration was 2×10^4^ cells/L. The test group was *G. aerucyinosum* co-cultured with *S. rhizophila* GA1 (final concentration was 1 × 10^5^ cells/mL). The total experimental cycle was 18 days. Error bars indicate the mean value ± standard deviation of three measurements of ChII *a*. (D) Analysis of AHLs from supernatant extracts of GA1 strain. AHLs extracted from cell-free culture supernatants were separated by thin-layer chromatography and detected using an overlay of agar seeded with *Agrobacterium tumefaciens* 136. Lane 1contains AHL standards (arrows point to C_8_-oxo-, C_10_-, and C_12_-AHL, respectively); lane 2 contains the *S. rhizophila* GA1 extracts.

In this study, an algicidal bacterium (*Stenotrophomonas rhizophila* GA1) was screened from the marine dinoflagellate. The morphology, optimal growth conditions and algicidal ability are shown in Fig. 1A–C. A summary of other data for the isolated strain is listed in Table 1.

**Table 1:** Basic information and genome features for *S. rhizophila* GA1.

Items	Descriptions
Geographic location	The coastal of Shenzhen, China
Latitude and longitude	N22°59′42.19″, E114°54′74.01″
Organism/strain	*Stenotrophomonas rhizophila* GA1
Gram strain	Negative
Cell shape	Rod
Color of colonies	Yellow
Temperature	16–36 °C
Optimal pH	8.0
Environment (biome)	Temperature, salinity, pH value, and sea biome
Environment (feature)	Water body of phycosphere (*G. aerucyinosum*)
Environment (Material)	Sea water
Sequencer	Illumina Hiseq. 2500
Data format	Processed
Experimental factor	Microbial strain
Experimental features	Whole genome sequence of *S. rhizophila* GA1
Consent	N/A
Assembly and annotation	CLC Genomics Workbench Version. 5.1
Finishing strategy	Primer design, PCR and sequencing
Genome size	5.92 Mb
GC content %	62.39%
Number of Contigs	67
Total contig size	5,929,188
Scafflods	8
Total scaffold size	6,598,546
Protein enconing genes	4579
tRNAs	64
rRNAs	26
Predicted AHL gene *LuxI* site	Contig 4
Encoding-AHL gene length	459–848 bp

**Fig. 2:**
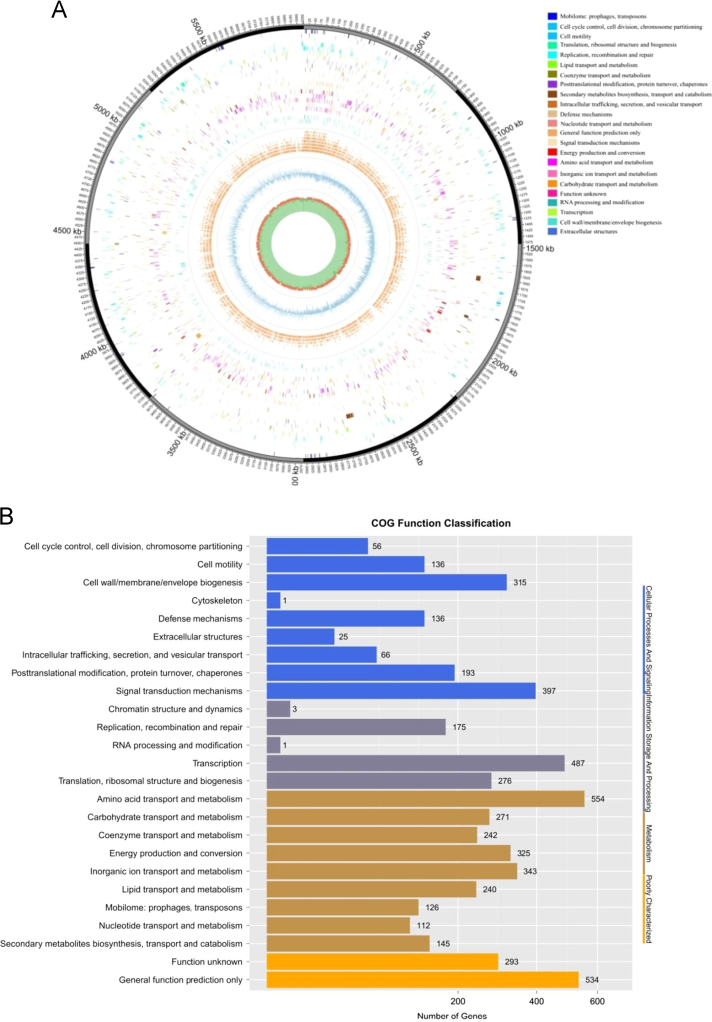
(A) Circular map for the whole genome of *S. rhizophila* GA1. From the outside to the center: encoding genes, predicted CDSs transcribed in the clockwise (or counter clockwise) direction, ncRNA, GC percent (%), and GC skew (G + C/G-C) in a 1000-bp window. (B) Functional category distribution of *S. rhizophila* GA1 (based on COG function statistics).

The whole genome of *S. rhizophila* GA1 contained 6,598,546 bases and a G+C content of 62.39% (Table 1). The analyses of the complete genome identified 4579 open reading frames. Homologous comparison by BLAST found 3395 CDS (coding sequence) involving 25 functional COGs (clusters of orthologous groups) and a part of the CDS involving 34 KEGG (Kyoto encyclopedia of genes and genomes) metabolic pathways (Fig. 2A).

Based on the functional categories of COGs and KEGG groups, 271 genes were involved in carbohydrate metabolism and 879 genes participated in amino acid transport and energy conversion (Fig. 2B). Several genes encoding putative AHL inducers ( LuxI-like proteins) were found in contig 4 of *S. rhizophila* GA1. The thin-layer chromatography (TLC) confirmed *S. rhizophila* GA1 strain has the ability to produce AHL signaling molecules (Fig. 1D). In addition, a chitinase-coding gene (*chi*) was found downstream of luxR; the protein encoded by this gene is believed to contribute to the ability of *S. rhizophila* GA1 to lyse its host (algae).

## 2 Experimental design, materials, and methods

### 2.1 Isolation

The seawater sample was collected from the dinoflagellate (*G. aerucyinosum*) bloom on the Shenzhen coast of China. An isolated strain GA1 caused significant algal lyse. The 16S rRNA gene sequence analysis revealed that it shared 99.7% similarity to the type strain of *Stenotrophomonas rhizophila*. We provisionally named this strain *S. rhizophila* GA1.

### 2.2 DNA extraction, sequencing, and assembly

Genomic DNA of *S. rhizophila* GA1 was extracted using the genomic DNA extraction kit (MoBio, CA, USA) following the protocols of the manufacturer. Whole-genome sequencing of the normalized DNA was performed using IlluminaHiseq. 2500 (San Diego, USA) instrument, as descripted in Glushchenko et al. [1]. De novo assembly was performed using CLC Genomics Workbench version 5.1 (CLC Bio, Denmark) and trimmed using a minimum Phred quality score of 20, a minimum length of 50 bp, allowing no ambiguous nucleotides and trimming off some low-quality nucleotides [2]. The reads were assembled with SOAPdenovo (V.2.04) [3], and the sequence was annotated using the RAST annotation server [4]. tRNA and rRNA genes were predicted by tRNAscan-SE [5] and RNAmmer [6], respectively. Genes were predicted using Glimmer 3.02 [7] and annotated by searching against the NCBI-nr and KEGG databases.

### 2.3 Thin-layer chromatography (TLC)

To verify the AHL-synthesizing activity of *S. rhizophila* GA1, reverse-phase thin-layer chromatography (TLC) was performed as described by Shaw et al. [8] and Ma et al. [9].

### 2.4 Data accessibility

The genome sequence data has been deposited in the GenBank database under accession numbers PRJNA485554.
